# miRNA-10a-5p Targeting the BCL6 Gene Regulates Proliferation, Differentiation and Apoptosis of Chicken Myoblasts

**DOI:** 10.3390/ijms23179545

**Published:** 2022-08-23

**Authors:** Genxi Zhang, Xinchao Zhang, Kaizhi Zhou, Xuanze Ling, Jin Zhang, Pengfei Wu, Tao Zhang, Kaizhou Xie, Guojun Dai

**Affiliations:** College of Animal Science and Technology, Yangzhou University, Yangzhou 225009, China

**Keywords:** miRNA-10a-5p, BCL6, myoblasts, proliferation, apoptosis, differentiation

## Abstract

Proliferation, differentiation, and apoptosis are three essential stages in cell development, and miRNAs can achieve extensive regulation of cellular developmental processes by repressing the expression of target genes. According to our previous RNA-seq results, miRNA-10a-5p was differentially expressed at different periods in chicken myoblasts, revealing a possible association with muscle development. In this study, we concluded that miRNA-10a-5p inhibited chicken myoblasts’ proliferation and differentiation and promoted chicken myoblasts’ apoptosis by directly targeting BCL6, a critical transcription factor involved in muscle development and regeneration. Overexpression of BCL6 significantly facilitated myoblasts’ proliferation and differentiation and suppressed myoblasts’ apoptosis. On the contrary, knockdown of BCL6 significantly repressed myoblasts’ proliferation and differentiation and induced myoblasts’ apoptosis. The results above suggest that miRNA-10a-5p plays a potential role in skeletal muscle growth, development and autophagy by targeting the BCL6 gene. We first revealed the functions of miRNA-10a-5p and BCL6 in the proliferation, differentiation, and apoptosis of chicken myoblasts.

## 1. Introduction

For animal husbandry, meat production of livestock is one of the important indicators to measure economic benefits. As one of the world’s most consumed kinds of meat, chicken is favored for its high protein, low price, and delicious taste. Along with the enhancement of life quality, the demand for chicken grows yearly, contributing to the urgent need to speed up the broiler breeding process. Nowadays, conventional breeding work has been slightly stretched by the generation interval. Therefore, more and more scholars attempt to explore the mechanism of muscle growth from the molecular level. Many experiments have been done to reveal how microRNAs (miRNAs) and their target genes function in the chicken myoblasts’ proliferation, differentiation, and apoptosis. miRNAs are a class of non-coding single-stranded RNA molecules of 18–24 nucleotides in length encoded by endogenous genes and are highly conserved across species. miRNAs can bind to the 3′ non-coding regions (3′ UTR) of mRNAs and then participate in post-transcriptional regulation of plant and animal organisms. This regulatory process plays an important role in cell proliferation, differentiation, apoptosis, invasion, and metastasis [1,2]. In addition to their functions within the cells that generate them, miRNAs can also exist in vesicles and act as a component of exosomes, enter the recipient cells through cytokinesis and inhibit gene expression within the recipient cells [3]. Many miRNAs are spatiotemporally specific, such as hsa-miR-8069 [4], mmu-let-7a [5] and gga-miRNA-27b-3p [6]. With the progress of research, more regulatory capabilities and modalities of miRNAs will be gradually discovered.

miRNA-10a-5p belongs to the miRNA10/100 family, consisting of five miRNAs: miR-10, miR-51, miR-57, miR-99, and miR-100. It has been reported that miR-10, miR-99, and miR-100 are highly conserved in several species. Simultaneously, these three miRNAs are located in the genome close to the tumor-related gene HOX and regulate the expression of the HOX gene, suggesting this miRNA family is inseparably related to tumorigenesis [7,8]. Currently, a large number of reports on miRNA-10a-5p focus on the differentiation, apoptosis, inflammation development, invasion, and migration of cancer cells. For example, in human ovarian cancer cells, miRNA-10a-5p inhibited the viability, colony formation, migration, and invasion ability of human ovarian cancer cells and down-regulated the expression of HOXA1. However, when HOXA1 was overexpressed, the inhibitory effect of miRNA-10a-5p on ovarian cancer cells was eliminated [9]. Except for this, a high level of miRNA-10a-5p in extracellular vesicles will induce prostate cancer. Hence, miRNA-10a-5p in extracellular vesicles can be used as a marker to detect prostate cancer [10]. miRNA-10a-5p is also involved in the process of fat production and accumulation. In goat intramuscular precursor adipocytes, miRNA-10a-5p inhibited the differentiation of intramuscular precursor adipocytes into adipocytes. In a similar way, miRNA-10a-5p inhibited cell differentiation and promoted cell conversion from the G1/G0 phase to the S phase in mouse precursor adipocytes, enhancing the proliferation of precursor adipocytes [11]. Until now, there has been no report about the effects of miRNA-10a-5p on skeletal muscle in livestock and poultry myoblasts.

B cell lymphoma 6 (BCL6) is a transcriptional inhibitor mainly expressed in germinal center B cells and lymphoma cells [12,13]. The protein encoded by BCL6 contains two distinct structural domains: the BTB/POZ structural domain and the zinc finger protein structural domain, which bind specifically to protein and DNA, respectively [14]. More and more studies have shown that BCL6 is ubiquitously distributed in different tissues and is widely involved in various life activities such as cell proliferation, differentiation, activation, apoptosis, DNA damage repair, and cell cycle regulation [15,16]. BCL6 is essential for developing classical dendritic cells in the spleen and plays a vital role in maintaining immune balance [17]. A study suggests that the expression of BCL6 is significantly down-regulated in the spleen tissue of F_1_ generation of chickens in Marek’s-disease-susceptible chickens [18]. In addition, BCL6 also functions in the sexual maturation of chickens. The expression of BCL6 in the hypothalamus is at a low level when chickens are not sexually mature, yet once sexually mature, its presentation will show a significant increase [19]. In mice, BCL6 can inhibit apoptosis of myogenic cells at the stage of differentiation [20], whereas the loss function of the BCL6 slightly promotes the activation of skeletal muscle satellite cells [21]. However, the effects of the BCL6 gene on the proliferation, differentiation, and apoptosis of chicken myoblasts have not been reported.

This study aimed to investigate the effects of miRNA-10a-5p on the chicken myoblasts’ proliferation, differentiation, and apoptosis and to elucidate the regulatory mechanisms of miRNA-10a-5p. This research will help in understanding the role of miRNAs in the growth and development of chicken skeletal muscle and to provide a theoretical basis for improving growth traits in chickens.

## 2. Results

### 2.1. miRNA-10a-5p Inhibits the Proliferation of Chicken Myoblasts

**Figure 1:**
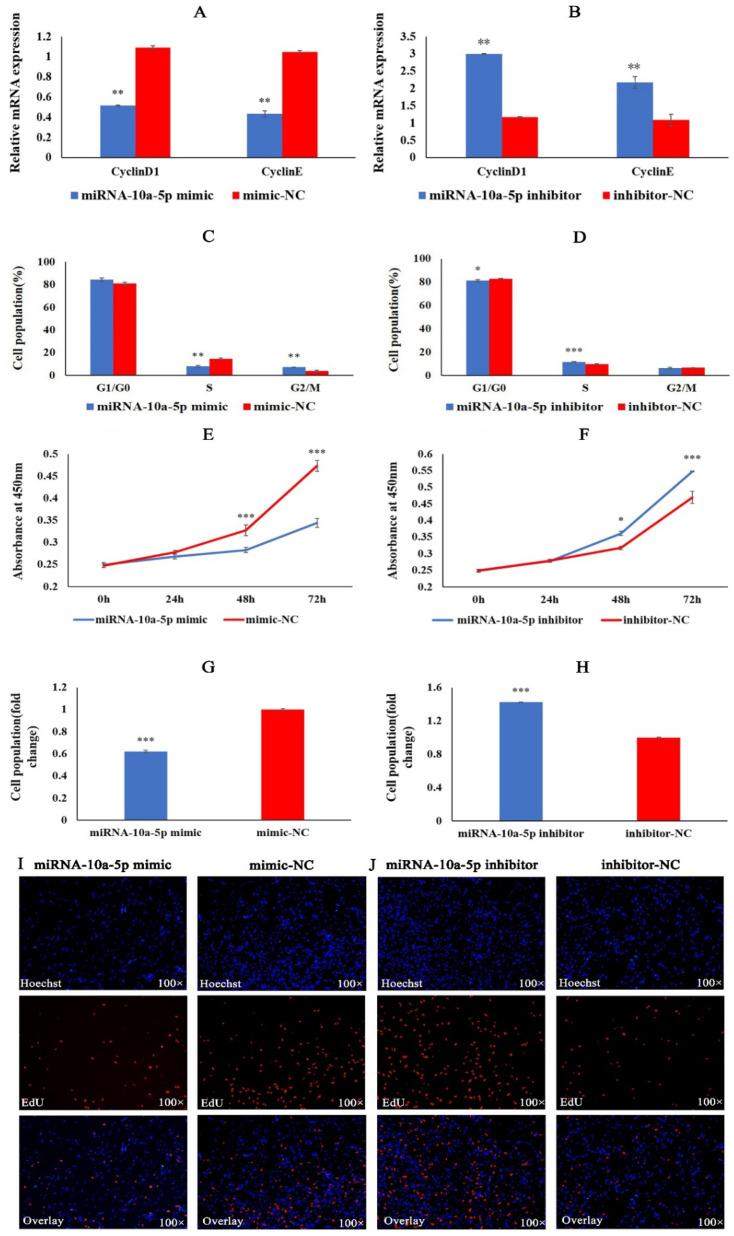
miRNA-10a-5p inhibits chicken myoblasts’ proliferation. (**A**,**B**) The relative mRNA expression of CyclinD1 and CyclinE after transfection with miRNA-10a-5p mimic and inhibitor in CMs. (**C**,**D**) The cell cycle changes by flow cytometry after transfection with miRNA-10a-5p mimic and inhibitor. (**E**,**F**) The absorbance values of cells at 450 nm detected by CCK8 after transfection with miRNA-10a-5p mimic and inhibitor. (**G**,**H**) Proliferation rates of CMs following transfection with miRNA-10a-5p mimic and inhibitor. (**I**,**J**) The image of cell proliferation under fluorescence inverted microscopy after transfection with miRNA-10a-5p mimic and inhibitor. In all graphs, the results are shown as mean ± SEM (standard error of the mean) (*n* = 3). * *p* < 0.05, ** *p* < 0.01, *** *p* < 0.001.

To understand the role of miRNA-10a-5p in the proliferation of chicken myoblasts, we transfected CMs with miRNA-10a-5p mimic, mimic-NC, miRNA-10a-5p inhibitor, and inhibit-N. QRT-PCR results showed that miRNA-10a-5p overexpression caused a significant decrease in the expression of CyclinD1 and CyclinE (Figure 1A). When transfected with miRNA-10a-5p inhibitor, a significant increase in the expression of CyclinD1 and CyclinE occurred (Figure 1B). Flow cytometry results showed that the number of cells in the G2/M phase significantly increased, and the percentage of cells in the S phase decreased significantly after miRNA-10a-5p overexpression (Figure 1C). The opposite results were obtained when the expression of miRNA-10a-5p was down-regulated (Figure 1D). The results of CCK8 assays showed that overexpression of miRNA-10a-5p inhibited the proliferation of myoblasts, whereas inhibition of miRNA-10a-5p promoted the proliferation of myoblasts (Figure 1 E,F). Finally, we analyzed cell viability using EdU. The cell viability of the miRNA-10a-5p overexpression group was significantly lower than that of the mimic-NC group. In contrast, the cell viability of the inhibition group was significantly higher than that of the inhibitor-NC group (Figure 1G–J). Based on the above results, we conclude that miRNA-10a-5p inhibits the proliferation of chicken myoblasts.

### 2.2. miRNA-10a-5p Inhibits the Differentiation of Chicken Myoblasts

**Figure 2:**
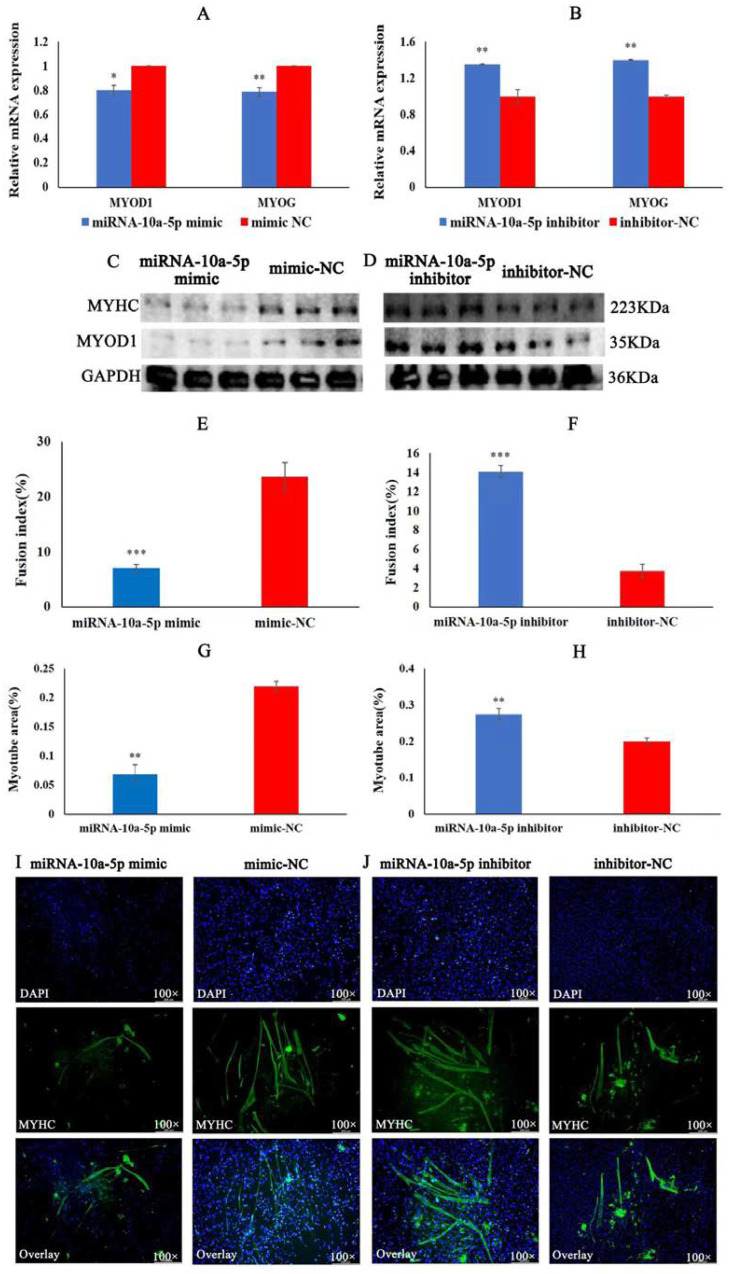
miRNA-10a-5p inhibits chicken myoblasts’ differentiation. (**A**,**B**) The qRT-PCR results of differentiation marker genes MYOD1 and MYOG after transfection with miRNA-10a-5p mimic and inhibitor. (**C**,**D**) The protein expression of MYHC and MYOD1 after transfection with miRNA-10a-5p mimic and inhibitor. (**E**,**F**) The fusion index (%) of chicken myoblasts after transfection with miRNA-10a-5p mimic and inhibitor. (**G**,**H**) The statistical results of the myotube area of myoblasts after transfection with miRNA-10a-5p mimic and inhibitor. (**I**,**J**) The image of IFA under fluorescence inverted microscope after transfection with miRNA-10a-5p mimic and inhibitor. In all graphs, the results are shown as mean ± SEM (standard error of the mean) (*n* = 3). * *p* < 0.05, ** *p* < 0.01, *** *p* < 0.001.

To explore the role of miRNA-10a-5p in the differentiation of chicken myoblasts, we performed qRT-PCR, Western blot, and IFA experiments on CMs in vitro by transfection of miRNA-10a-5p mimic and inhibitor. In the qRT-PCR experiment, the results showed that overexpression of miRNA-10a-5p significantly inhibited the mRNA expression of MYOD1 and MYOG. Meanwhile, the mRNA expression of MYOD1 and MYOG significantly increased after miRNA-10a-5p inhibition (Figure 2A,B). The Western blot showed that the protein expression of MYHC and MYOD1 was significantly lower in the miRNA-10a-5p mimic group than that in the mimic-NC group, whereas the protein expression of MYHC and MYOD1 was significantly higher in the miRNA-10a-5p inhibition group than that in the inhibitor-NC group (Figure 2C,D). Finally, the results of IFA showed that the myotube area and fusion index of the group transfected with miRNA-10a-5p mimic was significantly lower than that in the mimic-NC group (Figure 2E,G,I). There was a significant increase in myotube area and fusion index in the miRNA-10a-5p inhibition group compared to the inhibitor-NC group (Figure 2F,H,J). The findings above suggest that miRNA-10a-5p inhibits the differentiation of chicken myoblasts.

### 2.3. miRNA-10a-5p Promotes the Apoptosis of Chicken Myoblasts

**Figure 3:**
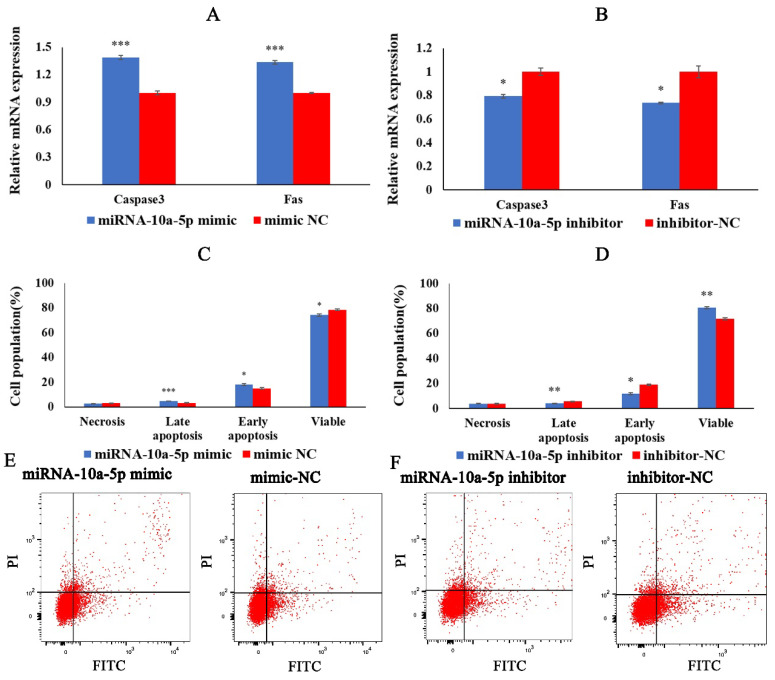
miRNA-10a-5p promotes chicken myoblasts’ apoptosis. (**A**,**B**) The relative expression of apoptosis marker genes Caspase3 and Fas by qRT-PCR after transfection with miRNA-10a-5p mimic and inhibitor. (**C**,**D**) Statistical chart of FCM test results. (**E**,**F**) The scatter plot of the FCM results after transfection with miRNA-10a-5p mimic and inhibitor. In all graphs, the results are shown as mean ± SEM (standard error of the mean) (*n* = 3). * *p* < 0.05, ** *p* < 0.01, *** *p* <0.001.

We examined the effect of miRNA-10a-5p on chicken myoblasts’ apoptosis by qRT-PCR and flow cytometry. We transfected miRNA-10a-5p mimics, miRNA-10a-5p inhibitor, mimic-NC, and inhibitor-NC into CMs. QRT-PCR results showed that the expression of apoptosis marker genes Caspase3 and Fas significantly increased 36 h after transfection with miRNA-10a-5p mimic, whereas the expression of Caspase3 and Fas decreased significantly in the group transfected with miRNA-10a-5p inhibitor (Figure 3A,B). In the flow cytometry results, the cells transfected with miRNA-10a-5p mimic showed significantly higher early and late apoptosis than those in the mimic-NC group. In contrast, the cells in the miRNA-10a-5p inhibition group showed significantly lower early and late apoptosis than those in the inhibitor-NC group (Figure 3C–E). The results above suggested that miRNA-10a-5p promotes the apoptosis of chicken myoblasts.

### 2.4. miRNA-10a-5p Has a Targeting Relationship with BCL6

**Figure 4:**
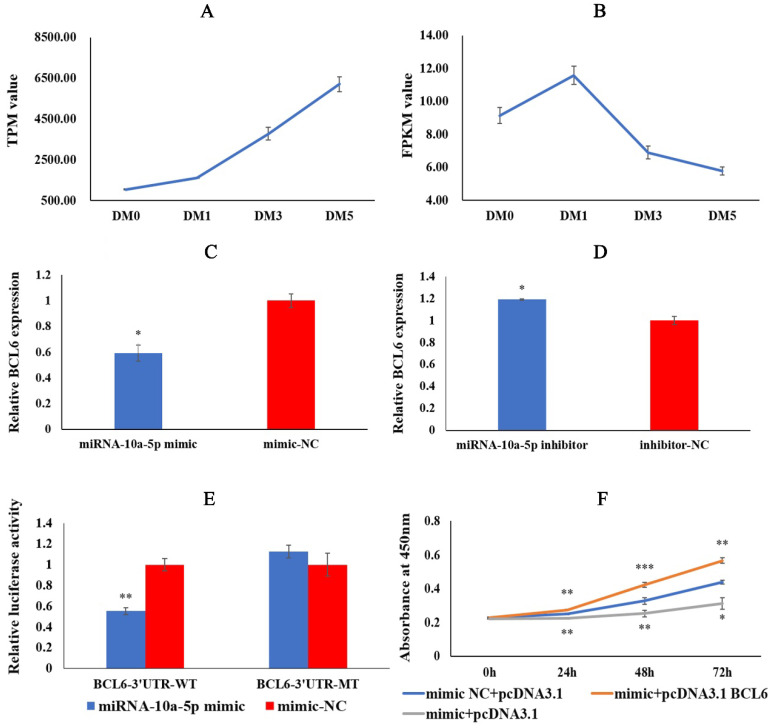
miRNA-10a-5p has a targeting relationship with BCL6. (**A**,**B**) DM0, DM1, DM3, and DM5 represent CMs that were induced to differentiate for 0, 24, 72, and 120 h, respectively. TPM is the abbreviation for Transcripts Per Million, and FPKM is the abbreviation for Fragments Per Kilobase per Million. (**C**,**D**) The relative expression of BCL6 after transfection with miRNA-10a-5p mimic and inhibitor. (**E**) The results of co-transfecting miRNA-10a-5p mimic and BCL6-3′UTR-WT; miRNA-10a-5p and BCL6-3′UTR-MT; mimic-NC and BCL6-3′UTR-WT; and mimic-NC and BCL6-3′UTR-MT in the dual-luciferase reporter assay. (**F**) Co-transfecting mimic-NC and pcDNA3.1; miRNA-10a-5p mimic and pcDNA3.1-BCL6; and miRNA-10a-5p and pcDNA3.1 in the rescue assay. In all graphs, the results are shown as mean ± SEM (standard error of the mean) (*n* = 3). * *p* < 0.05, ** *p* < 0.01, *** *p* <0.001.

The experiments above showed that miRNA-10a-5p played an important role in regulating chicken myoblasts’ proliferation, differentiation, and apoptosis. Therefore, we obtained the candidate target gene BCL6 using miRanda and miRBase, together with our previous RNA-seq data (Figure 4A,B). To verify whether BCL6 has a targeting relationship with miRNA-10a-5p, we detected the relative expression of BCL6 after miRNA-10a-5p overexpression and inhibition. The qRT-PCR result showed that the expression of BCL6 in the miRNA-10a-5p mimic group was significantly lower than that in the mimic-NC group, whereas transfection of miRNA-10a-5p inhibitor had the opposite result (Figure 4C,D). Dual-luciferase reporter assay showed that the miRNA-10a-5p mimic and pMIR-BCL6-3′UTR-WT co-transfected group had significantly lower luciferase activity in the mimic-NC and pMIR-BCL6-3′UTR-WT co-transfected group. The luciferase activity of the miRNA-10a-5p mimic and pMIR-BCL6-3′UTR-MT co-transfected group was not significantly different from that of the mimic-NC and pMIR-BCL6-3′UTR-MT co-transfected group (Figure 4E). These results revealed that miRNA-10a-5p should have a targeting relationship with BCL6. Finally, we co-transfected miRNA-10a-5p mimic + pcDNA3.1, miRNA-10a-5p mimic + pcDNA3.1-BCL6, and mimic-NC + pcDNA3.1 into CMs. The results showed that the absorbance values of the miRNA-10a-5p mimic + pcDNA3.1 group were significantly lower than those of the mimic-NC + pcDNA3.1 group. In contrast, the absorbance values of the miRNA-10a-5p mimic + pcDNA3.1-BCL6 group were significantly higher than those of the mimic-NC + pcDNA3.1 group (Figure 4F), which further indicated that miRNA-10a-5p had a targeting relationship with BCL6. All in all, BCL6 is a target gene of miRNA-10a-5p.

### 2.5. BCL6 Gene Promotes the Proliferation of Chicken Myoblasts

**Figure 5:**
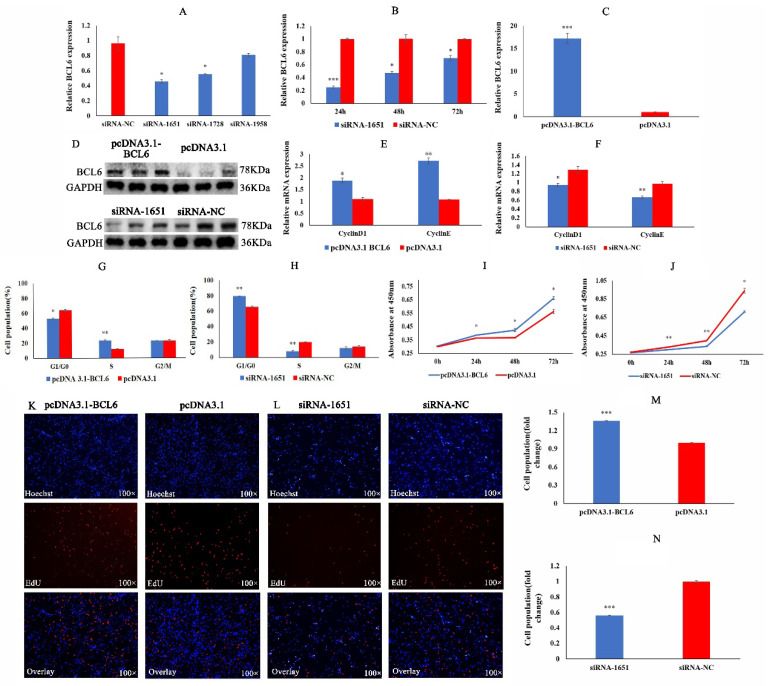
BCL6 gene promotes chicken myoblasts’ proliferation. (**A**) Optimal interference sequence screening. (**B**) The relative expression of BCL6 after transfection of pcDNA3.1-BCL6 and pcDNA3.1. (**C**) The optimal detection time after transfection of siRNA-1651. (**D**) The relative expression of BCL6 at the protein level after overexpression and inhibition of BCL6. (**E**,**F**) The expression of cycle-related genes CyclinD1 and CyclinE after transfection with pcDNA3.1-BCL6 and siRNA-1651. (**G**,**H**) Cell cycle changes detected by flow cytometry after transfection with pcDNA3.1-BCL6 and siRNA-1651. (**I**,**J**) The absorbance values of cells at 450 nm detected by CCK8 after transfection with pcDNA3.1-BCL6 and siRNA-1651. (**K**–**N**) The EdU results of cell proliferation after transfection with pcDNA3.1-BCL6 and siRNA-1651. In all graphs, the results are shown as mean ± SEM (standard error of the mean) (*n* = 3). * *p* < 0.05, ** *p* < 0.01, *** *p* <0.001.

To achieve the best interference effect, we screened out the best interference sequence (siRNA-1651) and the best interference detection time (Figure 5A,B). The overexpression efficiency of the overexpression plasmid pcDNA3.1-BCL6 was 16 times higher than that of the pcDNA3.1 group (Figure 5C). The detection results at the protein level showed that the interfering sequence and overexpression plasmid were also effective (Figure 5D). The experimental results above suggest that siRNA-1651 and pcDNA3.1-BCL6 can be used in subsequent experiments.

To understand the regulatory role of the BCL6 gene in the proliferation of CMs, we performed qRT-PCR, flow cytometry, CCK8, and EdU assays. QRT-PCR results showed that the expression of CyclinD1 and CyclinE was significantly increased after BCL6 overexpression, whereas the expression of CyclinD1 and CyclinE was significantly decreased after reduction of BCL6 activity (Figure 5E,F). Flow cytometry showed that overexpression of the BCL6 gene significantly increased the number of cells in the S phase, and cells in G1/G0 phase were significantly reduced. In contrast, reduction of BCL6 activity resulted in the opposite results (Figure 5G,H). It was clear from the effects of CCK8 that overexpression of the BCL6 gene increased the proliferative activity of CMs, whereas the reduction of BCL6 activity significantly inhibited the proliferative activity of CMs (Figure 5I,J). Finally, the EdU results showed that overexpression of the BCL6 gene increased the proliferative viability of CMs, whereas reduction of BCL6 activity diminished the proliferative viability of CMs (Figure 5K–N). These results suggest that the BCL6 gene promotes the proliferation of chicken myoblasts.

### 2.6. BCL6 Gene Promotes the Differentiation of Chicken Myoblasts

**Figure 6:**
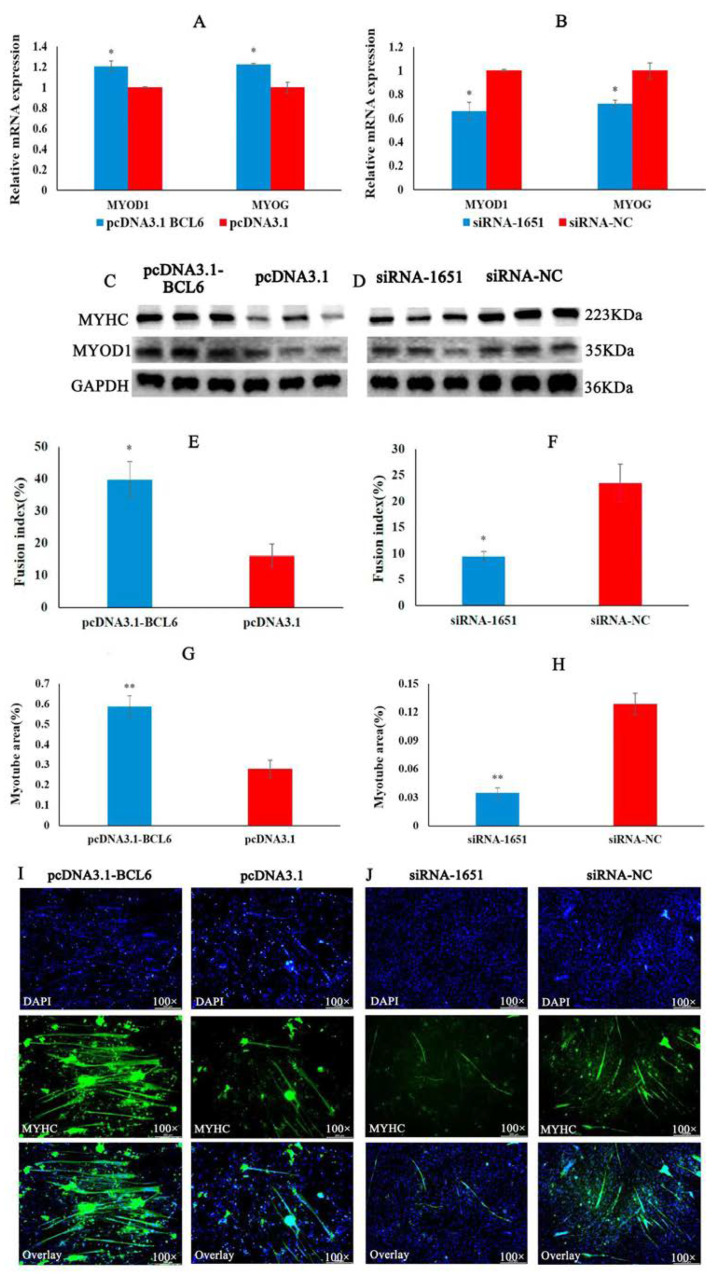
BCL6 gene promotes chicken myoblasts’ differentiation. (**A**,**B**) The results of qRT-PCR for differentiation marker genes MYOD1 and MYOG after transfection with pcDNA3.1-BCL6 and siRNA-1651. (**C**,**D**) The protein expression of MYHC, MYOD 1, and GAPDH after transfection with pcDNA3.1-BCL6 and siRNA-1651. (**E**,**F**) The fusion index (%) of chicken myoblasts after transfection with miRNA-10a-5p mimic and inhibitor. (**G**,**H**) The statistical results of the myotube area of myoblasts after transfection with pcDNA3.1-BCL6 and siRNA-1651. (**I**,**J**) The results of IFA after transfection with pcDNA3.1-BCL6 and siRNA-1651. In all graphs, the results are shown as mean ± SEM (standard error of the mean) (*n* = 3). * *p* < 0.05, ** *p* < 0.01.

We confirmed that miRNA-10a-5p inhibited the differentiation of CMs. Still, the role of its target gene, BCL6, in the differentiation of CMs was unclear, so we performed qRT-PCR, Western blot, and IFA experiments. In the qRT-PCR results, the relative expression of the differentiation marker genes MYOD1 and MYOG was significantly higher in the group transfected with pcDNA3.1-BCL6 than in that transfected with pcDNA3.1. The relative expression of MYOD1 and MYOG was significantly lower in the group transfected with siRNA-1651 than in that of siRNA-NC (Figure 6A,B). Western blot results showed that overexpression of BCL 6 increased the protein expression of MYHC and MYOD1, whereas inhibition of BCL 6 decreased the protein expression of MYHC and MYOD1 (Figure 6C,D). Finally, indirect immunofluorescence results showed that the overexpression of the BCL 6 gene significantly increased the myotube area and fusion index. Inhibition of the BCL6 gene yielded the opposite results to BCL6 overexpression (Figure 6E–J). Combined with qRT-PCR, Western blot, and IFA, we concluded that BCL6 promoted the differentiation of chicken myoblasts.

### 2.7. BCL6 Gene Inhibits the Apoptosis of Chicken Myoblasts

**Figure 7:**
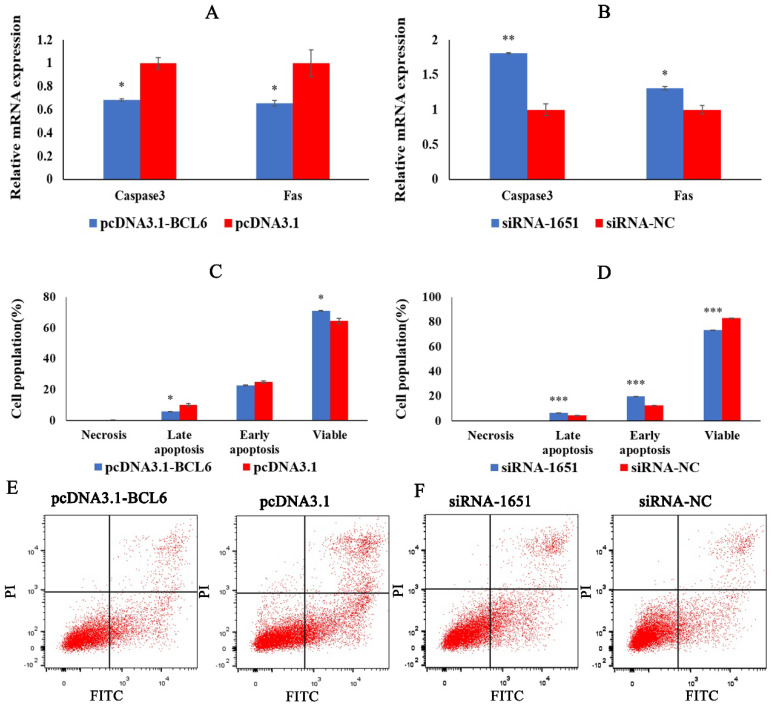
BCL6 gene inhibits chicken myoblasts’ apoptosis. (**A**,**B**) The relative expression of apoptosis marker genes Caspase3 and Fas after transfection with pcDNA3.1-BCL6 and siRNA-1651. (**C**,**D**) Statistical chart of FCM test results. (**E**,**F**) The scatter plot of the FCM results after transfection with pcDNA3.1-BCL6 and siRNA-1651. In all graphs, the results are shown as mean ± SEM (standard error of the mean) (*n* = 3). * *p* < 0.05, ** *p* < 0.01, *** *p* < 0.001.

To determine the role of BCL6 in the apoptotic process of CMs, we transfected pcDNA3.1-BCL6, pcDNA3.1, siRNA-1651, and siRNA-NC into CMs. We detected the expression of apoptosis marker genes Caspase3 and Fas, as well as apoptotic cells death. The results showed that the expression of Caspase3 and Fas was significantly reduced after the overexpression of the BCL6 gene (Figure 7A). In the flow cytometry results, overexpression of the BCL6 gene significantly inhibited late apoptosis cells and significantly increased the viable cells in CMs (Figure 7C,E). After inhibition of the BCL6 gene, the expression of Caspase3 and Fas significantly increased (Figure 7B). At the same time, early apoptosis and late apoptosis of CMs were significantly promoted, and the viable cells significantly decreased (Figure 7D,E). From the above results, we conclude that the BCL6 gene can inhibit the apoptosis of chicken myoblasts.

## 3. Discussion

Skeletal muscle is an essential part of the body, and its function is irreplaceable. The growth and development of skeletal muscle are influenced by multiple factors, ultimately leading to different skeletal muscle functions [22]. The miRNA–mRNA regulatory network is one of the most profound effects on growth and development. Current studies have shown that each miRNA in the human genome can interact with the mRNAs of more than one hundred target genes on average, and more than 60% of these interacting miRNA–mRNA binding sites are located in the 3′ UTR region [23]. In previous studies, miRNA–mRNA regulatory networks played an essential role in skeletal muscle growth and development of all stages as well as in the organisms [24,25]. Therefore, in this study, we investigated the role of miRNA-10a-5p and its regulation mechanism in the proliferation, differentiation, and apoptosis of chicken myoblasts during the proliferation and differentiation phases of development.

Cyclins are a family of proteins required to regulate the cell cycle through cyclin-dependent protein kinases (CDKs). CyclinD1 and CyclinE genes are essential genes regulating the transition of cells from the G1 to the S phase. In this process, CyclinD1 forms a protein complex with CDK4/CDK6, whereas CyclinE forms a protein complex with CDK2. Together, they drive the transition from the G1 phase to the S phase, thus promoting cell proliferation [26]. In mouse preadipocytes, miRNA-10a-5p up-regulated the expression of CyclinD, which decreased the proportion of cells in the G1 phase and increased the proportion of cells in the S phase, promoting cell proliferation [27]. In the study of Zhai et al., overexpression of miRNA-10a-5p decreased the number of cervical cancer cells that progressed to the S-phase and thus inhibited cell proliferation [28]. In addition, overexpression of miRNA-10a-5p in keratinocytes also inhibited cell proliferation [29]. Based on qRT-PCR, CCK-8, Edu, and Flow cytometry results, we concluded that miRNA-10a-5p could inhibit the proliferation of chicken myoblasts. 

Currently, many reports have indicated that miRNA-10a-5p overexpression inhibits the differentiation ability of cells. In vitro experiments, miRNA-10a-5p played a negative regulatory role in the osteogenic differentiation of human bone marrow MSCs by targeting ALP and RUNX2 genes. Similarly, in vivo miRNA-10a-5p negatively regulated the osteogenic differentiation of human bone marrow mesenchymal stem cells [30]. In our study, the effect of miRNA-10a-5p on chicken myoblasts’ differentiation was similar to that described above. miRNA-10a-5p can exert an inhibitory influence on chicken myoblasts’ differentiation. Interestingly, overexpression of miRNA-10a-5p inhibited the differentiation of neural stem cells toward astrocytes, but the differentiation of neural stem cells toward neural progenitors was not affected [31].

The balance of multicellular organisms is controlled not only by cell proliferation and differentiation but also by cell death. Fas, as a cell surface protein, is widely distributed in the cell membranes of immune cells such as thymocytes, activated T cells, B cells, and monocytes. When the Fas receptor on the cell surface receives the apoptotic signal delivered by Fas, it induces the release of intracellular Cytc. Cytc will form a complex with Apaf-1, Caspase 9 precursor, and ATP/dATP. The complex binds to the inactive caspase zymogen and eventually activates caspase 3, which triggers the caspase cascade reaction and brings apoptosis to an irreversible stage [32]. In the present study, the expression of Fas and Caspase3 genes was significantly increased after miRNA-10a-5p overexpression. The percentage of early and late apoptosis of cells obtained by flow cytometry was significantly increased, indicating that miRNA-10a-5p could promote the apoptosis of chicken myoblasts. When miRNA-10a-5p was disturbed, we got the opposite effect to that of overexpression. 

The BCL6 gene, a transcriptional regulator, is involved in the transcriptional regulation of more than 1200 genes and 14,000 proteins [33,34]. In vascular smooth muscle of hypertensive patients, overexpression of the BCL6 gene attenuated the expression of NADPH oxidase 4 (NOX4). It decreased the production of reactive oxygen species, inhibiting vascular smooth muscle cell proliferation [35]. However, in human trophoblast cells, BCL6 expression significantly promoted cell proliferation [36]. In our study, the BCL6 promoted the proliferation of chicken myoblasts. BCL6 also exerts different effects during cell differentiation according to the cell type. The BCL6 gene was discovered as an oncogene involved in the growth and development of B-cell lymphoma, which could contribute to the malignant phenotype by inhibiting DNA damage repair and blocking B-cell terminal differentiation [37]. In chicken bone marrow mesenchymal stem cells (MSCs), overexpression of the BCL6 gene inhibited the secretion of hematopoietic growth factors and blocked the differentiation of hematopoietic stem cells [33]. Expression of BCL6 in CD4+ T cells promoted follicular helper T cell (TFH) differentiation in mice [38,39]. 

The qRT-PCR, Western blot, and IFA results in our study showed that the BCL6 gene could promote the differentiation of chicken myoblasts. Pathway enrichment analysis of the target genes of the relevant miRNAs revealed that the BCL6 gene was enriched as a differentially expressed gene in the mTOR and FOXO signaling pathways, which are extremely closely related to apoptosis. Subsequently, the phenomenon that the BCL6 gene could inhibit cellular apoptosis was confirmed in the related functional validation [40]. In chicken hematopoietic stem cells, BCL6 also exerted an inhibitory effect on apoptosis [33]. In our study, the BCL6 gene inhibited the apoptosis of chicken myoblasts, which was consistent with previous studies. 

In many cases, miRNAs are involved in physiological or pathological stress, suggesting they function to exacerbate or protect the organism during pressure or disease. Although many skeletal muscle diseases differ in clinical and pathological manifestations, they all have a common feature of dysregulation of miRNA expression. Rao et al. [41] showed that miR-1 and miR-133a were drastically reduced in representative cell lines from each major rhabdomyosarcoma subtype (embryonal and alveolar), and mRNA targets of miR-1 and miR-133a are up-regulated in rhabdomyosarcoma. These results indicate the promise of enhancing rhabdomyosarcoma therapy using miRNAs as agents that mediate cytostasis and promote muscle differentiation. A similar study demonstrated that miR-1/206 suppressed c-Met expression in human rhabdomyosarcoma and could function as a potent tumor suppressor in c-Met-overexpressing tumors [42]. Our results indicated that miRNA-10a-5p and the target gene BCL6 have potential application value in the treatment of rhabdomyosarcoma and muscular atrophy. However, further research should be conducted before their application in the medical field.

## 4. Materials and Methods

### 4.1. Ethics Statement

All experimental animal protocols in this study were carried out in strict accordance with the “Jiangsu Province laboratory animal management measures”, and were approved by the Animal Ethics Committee of Yangzhou University (Approval number: Yzu DWLL-201903-001).

### 4.2. Cell Isolation, Culture, and Total RNA Extraction

The fertilized eggs of the Jinghai Yellow Chicken were supplied by Jiangsu Jinghai Poultry Group Co Ltd. and incubated in the Jiangsu Genetics and Breeding Laboratory. The isolation and culture of chicken primary myoblasts were carried out using the following method. After removing and quickly decapitating chicken embryos incubated until E12, the embryonic leg muscle tissue was taken to a sterile manipulative table. The isolated muscle was digested with collagenase type I (Gibco, Grand Island, NY, USA) at 37 °C to obtain single cells. The single cell suspension obtained after digestion was filtered through a cell strainer (Biosharp, Hefei, China) and centrifuged. The re-suspended cell suspension was subjected to three differential attachments in 10 cm cell culture dishes. Fibroblasts and blood cells were then removed. The growth medium was DME-F12 containing 20% fetal bovine serum and 1% penicillin-streptomycin-amphotericin. The differentiation medium was DME-F12 containing 2% fetal horse serum and 1% penicillin-streptomycin-amphotericin. All cells were cultured in a cell incubator (Binder, Tuttlingen, Germany) containing 5% CO_2_ at 37 °C. 

Total RNA was extracted from cells using the Trizol (Takara, Dalian, China) method and stored at −80 °C. The integrity of the RNA was examined by agarose gel electrophoresis. The quality and concentration of the RNA were measured by spectrophotometer (Thermo, Waltham, MA, USA).

### 4.3. Primer Design for Quantitative Real-Time PCR (qRT-PCR)

The design for miR-10a-5p and U6 primers was based on the stem-loop method using miRNA Design V1.01 software (Vazyme, Nanjing, China). The gene primers were designed using Premier Primer 5.0 software (Premier Biosoft International, Palo Alto, CA, USA). Meanwhile, primer sequences for MYHC, MYOD 1, and MYOG genes were referenced from those of Cai et al. [43]. All primers were synthesized by Sangon Biotech (Shanghai, China). The related information for miRNA and gene primers are shown in Table 1 and Table 2, respectively.

### 4.4. cDNA Synthesis and Quantitative Real-Time PCR (qRT-PCR)

The cDNA synthesis of miRNA was obtained using the miRNA 1st strand cDNA synthesis Kit (by stem-loop) (Vazyme, Nanjing, China). The expression of miRNA-10a-5p in cells was then detected using miRNA Universal SYBR qPCR Master Mix (Vazyme, Nanjing, China). The cDNA synthesis of the target gene was carried out using Hiscript QRT Supermax (Vazyme, Nanjing, China). Expression levels of relevant genes in cells were assayed using ChamQ SYBR qPCR Master Mix (Vazyme, Nanjing, China). A total amount of 500 ng RNA was used in the 10 μL reverse transcription system. And 2 μL cDNA was used for qRT-PCR in a 20 μL reaction system. QRT-PCR was done on an Applied Biosystems 7500 Fast DX real-time PCR instrument (ABI, Los Angeles, CA, USA). The reaction procedure of qRT-PCR was as follows: pre-denaturation at 95 °C for 30 s, denaturation at 95 °C for 10 s, and annealing at 60 °C for 30 s, 40 cycles. For the expression of genes, chicken β-actin was used as the housekeeping gene, whereas the housekeeping gene for miRNA expression was U6. Three replicates of each sample were tested. 

### 4.5. Sequence Synthesis and Vector Construction

miR-10a-5p mimic, mimic-NC (negative control), miR-10a-5p inhibitor, and inhibitor-NC were synthesized by RiboBio (Guangzhou, China). Small interfering RNAs (siRNAs) and siRNA negative control (siRNA-NC) for BCL6 knockdown and recombinant plasmid pcDNA3.1-BCL6 for overexpression of BCL6 were synthesized by GenePharma (Shanghai, China). Synthesis of recombinant plasmid included acquiring the BCL6 coding region (CDS) and inserting the CDS region into the pcDNA3.1 vector (Promega, Madison, WI, USA). The restriction sites of the recombinant plasmid are BamHI and EcoRI. Oligonucleotide sequences for overexpression and inhibition of miR-10a-5p are shown in Table 3.

The dual luciferase reporter vector was constructed by PCR amplifying the 3′ UTR regions containing the predicted binding sites of miR-10a-5p and BCL6. The amplified fragments were ligated into pMIR-REPORTER according to the homologous recombination method. The restriction enzyme cleavage sites for the recombinant plasmid are HindIII and MIuI. We sequenced the constructed recombinant plasmids to check whether the target fragment was successfully inserted. The pMIR-BCL6-3′UTR-MT vector was constructed by mutating the predicted binding sites from ACAGGGT to ACGTAGT according to the Mut Express II Fast Mutagenesis Kit V2 (Vazyme, Nanjing, China) instructions. Homologous recombinant primers for the BCL6 gene are shown in Table 4.

### 4.6. Cell Transfection and Dual-Luciferase Reporter Assay

All cells were transfected strictly according to the jetPRIME transfection reagent instructions (polyplus, Illkirch, France). 

DF-1 cells were cultured in DMEM (Sigma-Aldrich, St. Louis, MO, USA) medium containing 10% fetal bovine serum and 1% penicillin-streptomycin-amphotericin. DF-1 cells were uniformly inoculated in 24-well cell culture dishes. When the cell density reached 50%, pMIR-BCL6-3′UTR-WT and miR-10a-5p mimic; pMIR-BCL6-3′UTR-WT and mimic-NC; pMIR-BCL6-3′UTR-MT and miR-10a-5p mimic; and pMIR-BCL6-3′UTR-MT and mimic-NC were co-transfected into DF-1 cells. After 36 h of transfection, luciferase activity was measured on a multi-mode microtiter assay system (EnSpire, Perkin Elmer, USA) according to the Dual-Luciferase Assay Kit (Vazyme, Nanjing, China).

### 4.7. CCK-8 Assay and Edu Assay

Myoblasts were inoculated in 96-well cell culture plates. Cells were transfected when they reached a density of 40–60%. Cell proliferation was determined every 24 h after transfection by measuring absorbance at 450 nm with a multi-mode microtiter assay system (EnSpire, Perkin Elmer, USA) according to the instructions of the CCK-8 kit (Vazyme, Nanjing, China).

For the Edu assay, myoblasts were inoculated in 96-well cell culture plates. Cells were transfected when cell density reached 40–60%. After 36 h of transfection, the cells were processed according to the instructions of the Cell-Light^TM^ EdU Apollo567 In Vitro Kit (RiboBio, Guangzhou, China). Three random photographs were taken with an inverted fluorescent microscope (DMi8, Leica, Wetzlar, Germany). The total number of cells and the number of EDU positive cells were counted using ImagePro Plus 6.0 software (Media Cybernetics, Rockville, MD, USA).

### 4.8. Cell Cycle Assay

Myoblasts were inoculated in 12-well cell culture plates. When the cells reached the density of approximately 60%, the cells were transfected. After 36 h of transfection, cells were collected in 1.5 mL EP tubes. One ml of pre-cooled 70% ethanol was added to each tube, and cells were fixed at 4 °C for 24 h. Finally, 500 μL of propidium iodide (50 ug/mL, TianGen, Beijing, China) was added to each cell sample for staining and incubated at 37 °C for 30 min, protected from light. Cells were analyzed on a FACSAria SORP flow cytometer (BD Inc. Franklin, NJ, USA). Data analysis was performed using FlowJo V10 software (Becton, Dickinson and Company, New York, NY, USA).

### 4.9. Apoptosis Assay

Cell inoculation and transfection were consistent with the detection of the expression of the apoptosis marker genes. Three replicates were set for each group. After 36 h of transfection, the cells were treated according to the instructions of the apoptosis assay kit (Solarbio, Beijing, China) and detected by flow cytometry. Finally, the data were statistically analyzed using FlowJo V.10 software to plot the cell distribution.

### 4.10. Indirect Immunofluorescence Assay (IFA)

Myoblasts were inoculated in 12-well cell culture plates, and when the cell density reached 70%, the growth medium was replaced with a differentiation medium, followed by cell transfection. Myotube differentiation was observed at 72 h. When many myotubes appeared, they were washed with PBS (Solarbio, Beijing, China) and then fixed in 4% paraformaldehyde (Solarbio, Beijing, China) for 30 min. Immediately afterward, the cells were permeated with PBS containing 0.5% Triton X-100 (Solarbio, Beijing, China) for 30 min. After permeabilization, the cell samples were blocked with PBS containing 10% goat serum for 1 h in a 37 °C incubator. At the end of blocking, the goat serum was removed, and 500 μL of primary antibody (MYHC, Proteintech, Wuhan, China. 1:500) was added to each well. The samples were incubated in a shaker at 4 °C for 24 h. 

At the end of incubation, the samples were washed with 1× TBST (Solarbio, Beijing, China). A total of 500 μL of secondary antibody (FITC-IgG, Bioss, Beijing, China, 1:400) was then added and incubated for 1 h at 37 °C protected from light. After incubation with the secondary antibody, the secondary antibody was removed, and the samples were washed with 1 × TBST. A total of 500 μL of DAPI (Beyotime, Shanghai, China) staining solution was added. The cell samples were incubated for 5 min at room temperature, protected from light, followed by aspiration of DAPI and washing of the cells with 1 x TBST. Finally, an anti-fluorescence quencher (Beyotime, Shanghai, China) was added to each well. Myotubes were observed and photographed on an inverted fluorescent microscope (DMi8, Leica, Wetzlar, Germany). The sample photographs were processed with ImagePro Plus 6.0, and the percentage of the total area covered by the myotubes was calculated and used as the myotube area. The fusion index was calculated as a percentage of nuclei inside the Desmin positive myotubes relative to the total number of nuclei.

### 4.11. Western Blot Assay

Myoblasts were inoculated in 6-well cell culture plates. When the cell density reached 60%, the differentiation medium containing 2% fetal horse serum was replaced, and the cells were subsequently transfected. After 36 h of transfection, the culture medium was removed, and cells were washed with PBS. Then 150 μL of Western and IP cell lysate (containing PMSF at a concentration of 1 mM) was added to each well. The cell samples were fully lysed, collected into 1.5 mL EP tubes, and centrifuged at 12,000 r/min for 5 min, and the supernatant was collected with a new 1.5 mL EP tube. After detecting the protein concentrations using the BCA Protein Quantification Kit (Vazyme, Nanjing, China), the protein samples were diluted to the same concentration. Then, 5 × SDS-PAGE Loading Buffer (Solarbio, Beijing, China) was added to 1/4 volume of the protein samples, mixed, and boiled for 10 min (100 °C) in a metal bath. After cooling to room temperature and centrifuging at 3000 r for 2 min, the supernatant was used for Western blot. 

The protein samples were subjected to polyacrylamide gel electrophoresis (GenScript, Nanjing, China). It was then transferred to PVDF (BIO-RAD, Hercules, CA, USA). The PVDF membranes were blocked with 5% BSA for 1 h at room temperature. Then the primary antibody was added and incubated overnight at 4 °C. PVDF membranes were washed with 1 × TBST (Solarbio, Beijing, China), incubated with secondary antibodies for 1 h at room temperature, and blotted for display by coagulation chemiluminescence (ECL). Finally, the relative expression of the proteins was obtained by the ChemDocTMTouch imaging system (Bio-Rad, Hercules, CA, USA). The antibodies and their dilution ratios were as follows: MYHC rabbit polyclonal antibody (25182-1-AP, Proteintech, Wuhan, China, 1:500), BCL6 rabbit polyclonal antibody (bs-2734R, Bioss, Beijing, China, 1:500), GAPDH rabbit polyclonal antibody (ET1601-4, HUABIO, Hangzhou, China, 1:500), and HRP-binding Mouse anti-rabbit IgG (bsm-33179M-HRP, Bioss, Beijing, China, 1:2000).

### 4.12. Statistical Analysis

The relative expression of miRNA-10a-5p and related genes was calculated by the 2^−ΔΔCt^ method. The results were compared between the experimental and the control groups using the unpaired Student’s t-test with SPSS 25.0 (SPSS Inc., Chicago, IL, USA). Where *p* < 0.05 is marked as “*”, *p* < 0.01 is marked as “**” and *p* < 0.001 is marked as “***”.

## 5. Conclusions

In this study, we reveal that miRNA-10a-5p represses chicken myoblasts’ proliferation and differentiation and promotes chicken myoblasts’ apoptosis. As the target gene of miRNA-10a-5p, BCL6 exhibits an active role in the proliferation and differentiation of myogenic cells and impedes apoptosis. The mechanism of miRNA-10a-5p binding to BCL6 in regulating skeletal muscle development helps improve the regulatory network of myoblast proliferation, differentiation, and apoptosis. It provides a solid theoretical foundation for the practice of molecular breeding.

## Figures and Tables

**Table 1:** miRNA primers used for qRT-PCR.

Name	Primer Sequence (5′-3′)	Annealing Temperature (°C)
Gga-miRNA-10a-5p	F: CGCGTACCCTGTAGATCCGA R: AGTGCAGGGTCCGAGGTATT	60
U6	F: GTCACTTCTGGTGGCGGTAA R: GTTCAGTAGAGGGTCAAA	60
Stem-loop primer	GTCGTATCCAGTGCAGGGTCC GAGGTATTCGCACTGGATACGAC	

**Table 2:** Gene primers used for RT-PCR.

Gene	Primer Sequence (5′-3′)	Product Size(bp)	Annealing Temperature (°C)	Accession Number
BCL6	F: CTCTGGCTCAGCCTTTGGA R: GCACTTGTAGGGTTTGTCGC	169	60	NM_00102930.1
β-actin	F: CAGCCATCTTTCTTGGGTAT R: CTGTGATCTCCTTCTGCA TCC	169	60	NM_205518.1
MYOD 1	F: GCTACTACACGGAATCACCAAAT R: CTGGGCTCCACTGTCACTCA	200	60	NM_204214.2
MYOG	F: CGGAGGCTGAAGAAGGTGAA R: CGGTCCTCTGCCTGGTCAT	320	60	NM_204184.1
Caspase3	F: TGGCCCTCTTGAACTGAAAG R: TCCACTGTCTGCTTCAATACC	139	60	NM_204725.1
Fas	F: TCCACCTGCTCCTCGTCATT R: GTGCAGTGTGTGTGGGAACT	78	60	NM_001199487.1
Cyclin D1	F: CTGCTCAATGACAGGGTGC R: TCGGGTCTGATGGAGTTG	341	60	NM_205381
CyclinE	F: ACCTAAAATGAGAACAATCC R: GGCAACAATACCTCCTAAA	381	60	NM_001031358.2

**Table 3:** Oligonucleotide sequences for overexpression and inhibition.

Fragment Name	Sequence(5′-3′)
miRNA-10a-5p mimic	UACCCUGUAGAUCCGAAUUUGU AAAUUCGGAUCUACAGGGUAUU
mimic-NC	UUCUCCGAACGUGUCACGUTT ACGUGACACGUUCGGAGAATT
miRNA-10a-5p inhibitor	ACAAAUUCGGAUCUACAGGGUA
inhibitor-NC	CAGUACUUUUGUGUAGUACAA
siRNA-1651	GCGAGAACGGAGCCUUCUUTT AAGAAGGCUCCGUUCUCGCTT
siRNA-1727	GCAAGUCCACAGCGACAAATT UUUGUCGCUGUGGACUUGCTT
siRNA-1968	GCUCAUGUGCUCAUUCAUATT UAUGAAUGAGCACAUGAGCTT
siRNA-NC	UUCUCCGAACGUGUCACGUTT ACGUGACACGUUCGGAGAATT

**Table 4:** Homologous recombinant primers for the BCL6 gene.

Primer Name	Primer Sequence	Product Size (bp)	Annealing Temperature (°C)
BCL6-3′UTR-WT	F:AAAGATCCTTTATTAAGCTTTTTTCCCCAGTCTTATTT R:ATAGGCCGGCATAGACGCGTTTGGCGCTTCACTTTTCT	143	62
BCL6-3′UTR-MT	F:CGGGAGACGTAGTTTGGGCTGTGTCTAAACTGCAT R:CAAACTACGTCTCCCGAGATTGTTTATAACACAAGC	6581	65

## Abbreviations

3′ UTR: 3′ untranslated region; CMs: chicken myoblast; CDS: coding domain sequence; WT: wild type; MT: mutant type.
